# Analysis of High-Dimensional Coordination in Human Movement Using Variance Spectrum Scaling and Intrinsic Dimensionality

**DOI:** 10.3390/e27040447

**Published:** 2025-04-21

**Authors:** Dobromir Dotov, Jingxian Gu, Philip Hotor, Joanna Spyra

**Affiliations:** 1 Department of Biomechanics, University of Nebraska Omaha, Omaha, NE 68182, USA; 2 Computer Science Department, University of Ghana, Legon, Accra P.O. Box LG 581, Ghana; 3 Independent Researcher, Omaha, NE 68182, USA; spyraj@gmail.com

**Keywords:** biomechanics, collective dynamics, coordination dynamics, dimensionality reduction, Kuramoto model, movement science, multivariate synchronization analysis

## Abstract

Full-body movement involving multi-segmental coordination has been essential to our evolution as a species, but its study has been focused mostly on the analysis of one-dimensional data. The field is poised for a change by the availability of high-density recording and data sharing. New ideas are needed to revive classical theoretical questions such as the organization of the highly redundant biomechanical degrees of freedom and the optimal distribution of variability for efficiency and adaptiveness. In movement science, there are popular methods that up-dimensionalize: they start with one or a few recorded dimensions and make inferences about the properties of a higher-dimensional system. The opposite problem, dimensionality reduction, arises when making inferences about the properties of a low-dimensional manifold embedded inside a large number of kinematic degrees of freedom. We present an approach to quantify the smoothness and degree to which the kinematic manifold of full-body movement is distributed among embedding dimensions. The principal components of embedding dimensions are rank-ordered by variance. The power law scaling exponent of this variance spectrum is a function of the smoothness and dimensionality of the embedded manifold. It defines a threshold value below which the manifold becomes non-differentiable. We verified this approach by showing that the Kuramoto model obeys the threshold when approaching global synchronization. Next, we tested whether the scaling exponent was sensitive to participants’ gait impairment in a full-body motion capture dataset containing short gait trials. Variance scaling was highest in healthy individuals, followed by osteoarthritis patients after hip replacement, and lastly, the same patients before surgery. Interestingly, in the same order of groups, the intrinsic dimensionality increased but the fractal dimension decreased, suggesting a more compact but complex manifold in the healthy group. Thinking about manifold dimensionality and smoothness could inform classic problems in movement science and the exploration of the biomechanics of full-body action.

## 1. Introduction

Stable and efficient movement over various terrains is a skill that was necessary for our survival as a species throughout the evolutionary time scale and is essential for independent daily living on the time scale of an individual lifespan. To achieve upright bipedal locomotion, the human body needs to organize a system consisting of many biomechanical degrees of freedom. Traditionally referred to as the Bernstein’s degrees of freedom question (foundational in human movement science, motor control, and related branches of neuroscience) [1], the challenge is to understand how the nervous system resolves the redundancy of biomechanical degrees of freedom while ensuring context sensitivity to environmental and task constraints [2]. The role of hierarchical, heterarchical, and coalitional approaches to organization have been discussed in this context [3,4]. A more recent approach has been to reframe the redundancy as a bliss of motor abundance, because spare resources can be used to achieve adaptive properties such as stability, efficiency, and resistance to perturbations [5,6].

This theoretical problem can be illustrated in terms of its implications for the acquisition of motor skills [7]. For example, a beneficial strategy for beginning to learn how to throw a basketball appears to be to lock all degrees of freedom but one and use it to complete the desired trajectory. This is the *freezing* stage of learning. With continued learning, one begins to unlock degrees of freedom so that they make complementary contributions to the throw. This is the *release stage*. To achieve skilled performance, players are often instructed that the shot should fluidly progress from the feet all the way to the wrist and fingers. Indeed, evidence confirms that center of mass (related to the body’s posture) and ball release variables (how and when the ball leaves the hand) are coordinated but not locked to each other in skilled throws [8]. Despite its intuitive nature, the freeze–release principle could be described as anecdotal because it is not supported conclusively by the overall evidence in the literature [9]. This could be due to past reliance on small numbers of focal kinematic and kinetic variables instead of on a comprehensive analysis of full-body movement.

It is a remarkable fact that full-body coordination has received relatively less attention than single pairs of coordinating segments. This is despite the foundational status of the degrees of freedom problem and the inconclusive results in associated problems such as coordination during development and skill acquisition. A PubMed literature searches (https://pubmed.ncbi.nlm.nih.gov/?term=((coordination+dynamics)+AND+(human)+AND+(movement))+AND+((multivariate)+OR+(multi-variate)+OR+(multidimensional)+OR+(dimensionality+reduction)) and https://pubmed.ncbi.nlm.nih.gov/?term=((coordination+dynamics)+AND+(human)+AND+(movement)) accessed on 19 March 2025) with the terms ((coordination dynamics) AND (human) AND (movement)) AND ((multivariate) OR (multi-variate) OR (multidimensional)) returned 74 papers, but with the terms ((coordination dynamics) AND (human) AND (movement)), the number was 3395. One historical reason for this blind spot has been the complexity and cost of high-density motion tracking techniques. More recently, various forms of motion tracking technology, including markerless motion tracking, have become practical. Furthermore, the ubiquity of data-sharing practices has made it cost-effective to develop computational techniques using publicly available datasets, an approach that has enabled tremendous progress in other domains such as machine learning and AI. The time is ripe to breathe new life into classical theoretical questions in movement science.

The remaining obstacle in this context has been the lack of analytical tools, with few exceptions such as the human movement kinectome [10], generalized cross-wavelet transform [11], self-organizing maps [12], and multivariate Lyapunov exponent [13]. Some multivariate tools tend to be extensions of uni- and bi-variate methods for coordination dynamics. It can be expensive to scale these to dozens or even hundreds of variables because of computational and theoretical limitations. Any method that begins by parameterizing each pairwise relationship will have to exhaust all possible pairs. The number of these pairs, and the corresponding computational time and parameter space, accelerates with the number of dimensions. The family of methods based on recurrence quantification analysis, popular in the movement sciences, have been extended first to two, and then to more variables [14]. This comes at a cost, though, as it requires estimating parameters for each input variable. Furthermore, extending a method to receive more variables does not always extend its interpretation. For example, the order parameter *R* generalizes the pairwise relative phase to collectives of oscillators, yet it fails to distinguish between scenarios of uniformly poor synchronization among all oscillators and scenarios of strong but highly clustered synchronization. The cluster phase method is an extension of the order parameter [15]. Its limitation is that it addresses scenarios with one cluster only, the mean field. This limitation can be avoided by a combined approach involving the analysis and numerical modeling of coupled oscillators [16]. This is possible in scenarios when the individual dynamics are known to a reasonable degree, such as when each unit can be approximated by an oscillator. Another innovative approach is to determine topological properties of the patterns of change in all relative phases in a collective [17]. In the following, we propose an interpretable and inherently multivariate analysis of high-density coordination based on estimating manifold properties.

### From Dimensionality Reduction to Manifold Smoothness

We propose to use a method for measuring and interpreting geometrical properties of manifolds embedded in high-dimensional time-series data. A manifold is defined as a *d*-dimensional surface that curves and loops on itself as it is embedded in an *n*-dimensional observation space. A typical first step when dealing with such data is to apply a dimensionality reduction method to remove redundancies shared between variables. Indeed, principal component analysis (PCA) can be used in the study of full-body movement kinematics to help focus the analysis on relevant patterns of movement [18,19]. PCA is a dimensionality reduction technique that projects a given dataset into a new coordinate system such that each axis, called a principal component (PC), explains as much variance in the original data as possible while staying orthogonal to the other axes. Intuitively, PCA can be thought of as taking a camera that is pointing at a cloud of data points and turning it in such a way that the data line up in a useful axis, like looking at a tree from the top versus the side. Dimensions containing matching correlated patterns line up relative to the camera and are seen as one, and unique patterns are made more visible by projecting them on unique axes to emphasize their variance. Importantly, PCs are ranked by the amount of variance they explain. In this way, the first PCs contain gross features of the data and subsequent PCs contain ever finer details, until only noise is left. PCA has been widely used in multiple fields as a way of compressing information by retaining only the top PCs, for testing to what extent information is subject to compression, or for emphasizing unique information.

It is less often appreciated that PCA can be used not only for reducing dimensionality but also for measuring geometric properties of the lower-dimensional manifold [20]. The rate at which the variance (explanatory power) decreases over consecutive PCs is related to the fractal geometry of the manifold on which the original data live. The decay rate of the so-called variance spectrum or eigenspectrum is bounded by the smoothness and dimensionality—independently—of the unique manifold embedded in the high-dimensional observation space. The decay in the variance spectrum is given by the following scaling law [20]:

(1) $$s_{n}\propto n^{-\alpha }$$

**Figure 1:**
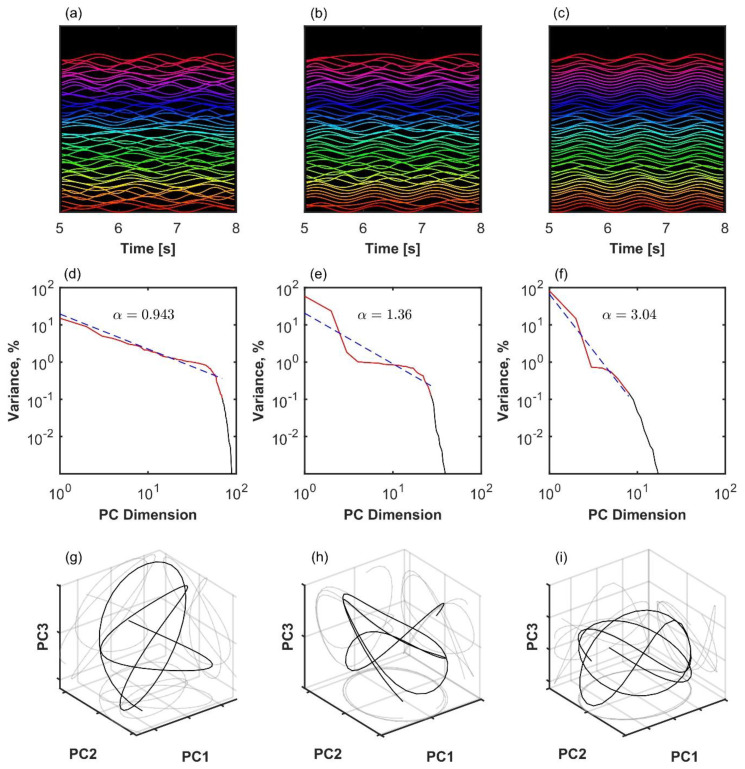
Variance spectrum analysis applied to three examples of the finite size Kuramoto system with *N* = 100 oscillators and different levels of coupling. Left column (**a**,**d**,**g**): weak coupling at the beginning of the critical region, *K* = 2. Middle column (**b**,**e**,**h**): moderate coupling in the middle of the critical region, *K* = 2.7. Right (**c**,**f**,**i**): strong coupling close to global synchronization, *K* = 4. (**a**–**c**): Time series of simulated oscillators. A sparse sample of oscillators shifted on the *y*-axis and in a short time window are shown for visibility. (**d**–**f**): Eigenspectrum scaling computed by fitting an inverse power law $s_{n}\propto n^{-\alpha }$ (red line) to the variance in principal components (black). In the first two columns (**a**,**d**,**g** and **b**,**e**,**h**), the observed scaling was α<3, consistent with the lack of global synchronization and manifold d>1, see Equation (2). (**g**–**i**): The first three principal component projections of the oscillator collectives.

If the original data live on a smooth manifold, meaning that the manifold is differentiable everywhere, then its variance spectrum must decay faster than or equal to a threshold,

(2) $$\alpha_{critical}=1+\frac{2}{d}$$

where *d* is the dimension of the manifold (see Figure 1d–f). For d→∞, this becomes the familiar power law $s\propto n^{-1}$ arising in various forms in one-dimensional time series. The relationship between the scaling properties of one-dimensional time series, a widely explored form of analysis in movement science [21,22], and the scaling properties of high-dimensional embedding data, is yet to be investigated.

The relationship between observation space, the scaling of the principal component variance spectrum, manifold dimension, and manifold differentiability was explained in the context of information encoding in neural space consisting of thousands of neurons [20]. Here, we will give it a biomechanical interpretation. To improve the coding efficiency of motor control, it would be convenient to distribute separate aspects of the movement to independent degrees of freedom. For example, early robotics designs often had no redundancy among their joints and motors [23]. In the variance spectrum space, this would result in a low α, with many small principal components with little difference among their eigenvalues, i.e., a slowly decreasing eigenspectrum. This would likely violate the manifold smoothness condition, as the trajectory would be jumping discretely from one dimension of the manifold to another. On the opposite end, control could be simplified by locking degrees of freedom to each other. In the variance spectrum space, this would result in a high α: few principal components account for all variance and eigenvalues decay very fast. Our hypothesis is that biomechanical systems are positioned in an intermediary range, above, but close to, the critical scaling, Equation (2). In this range, the underlying manifold is differentiable; hence, movement is efficiently smooth but still distributed among degrees of freedom. In the following section, we illustrate these ideas using simulated Kuramoto systems of oscillators as minimal examples of systems with phase-locking among its variables. Then, we test the hypothesis by analyzing full-body gait kinematics recorded in human participants with varying levels of motor disability.

Note that the objective of the present approach is not to recover the original components by mapping them to unique components of the PCA, which is a linear method. As empirical data often contain nonlinear features, there are a variety of methods for the nonlinear dimensionality reduction, each of which is suitable for different scenarios, depending on the purpose of the analysis and the type of nonlinearity [24]. In this work, we resort to nonlinear methods for estimating the fractal dimension of the data.

There is abundant literature on the coordination of neural dynamics involved in motor behavior [25] and generally on the dimensionality reduction of neural data [26]. Different modalities of observation and modeling confirm the presence of low-dimensional high-amplitude modes embedded inside high-dimensional neural dynamics during motor tasks [27,28,29,30,31]. Interestingly, this work long preceded the study of the dimensionality of full-body kinematics as it is addressed here. The neural substrate of movement organization is a large field; however, it deserves special attention because neural data pose their own challenges beyond the present scope.

## 2. Materials and Methods

### 2.1. Finite Size Kuramoto System Around the Critical Region of Coupling Strength

The Kuramoto dynamic system of coupled phase oscillators, Equation (3), was conceptualized as a model of a coupling-dependent spontaneous transition to a globally ordered state exhibited by populations of dynamic units with different natural frequencies.

(3) $${\dot{\theta }}_{i}=\omega_{i}+\frac{K}{N}\sum_{j=1}^{N}\sin\left(\theta_{j}-\theta_{i}\right)$$

Here, $\theta_{i}$ is the phase of oscillator i, each has a preferred frequency $\omega_{i}$ describing how fast around the unit circle it likes to go, K is the coupling strength, and N is the number of oscillators. Finite size and finite time simulations do not necessarily reflect the stable analytical properties of the ideal model [32,33]. Nevertheless, they exhibit their own interesting dynamic phenomena [34,35], and have provided model-based insight about synchronization in a large variety of natural systems (for some examples, [36,37,38]).

The model is suitable for testing the relationship between redundancy, manifold dimension, and variance scaling in a system of dynamic variables with varying synchronization, ranging from total independence to total convergence to a smooth global manifold with d=1. This is because the model gives a mathematical account of how individual oscillators become enslaved by the mean field. This can be seen by using the definition of the mean field of phases, Equation (4), to express Equation (3) equivalently in terms of the coupling between individual oscillators and the mean field. In Equation (5), ψ is the mean field phase. Global synchronization, the coherence among oscillators, is measured by the mean field amplitude r, which is also called the order parameter.

(4) $${re}^{i\Psi }=\frac{1}{N}\sum_{j=1}^{N}e^{i\theta_{j}}$$

(5) $${\dot{\theta }}_{i}=\omega_{i}+rK\sin\left(\Psi -\theta_{i}\right)$$

We assumed that the oscillators are also subject to Gaussian random variation $N\left(0,\sigma \right)$.

(6) $${\dot{\theta }}_{i}=\omega_{i}+\frac{K}{N}\sum_{j=1}^{N}\sin\left(\theta_{j}-\theta_{i}\right)+N\left(0,\sigma \right)$$

**Figure 2:**
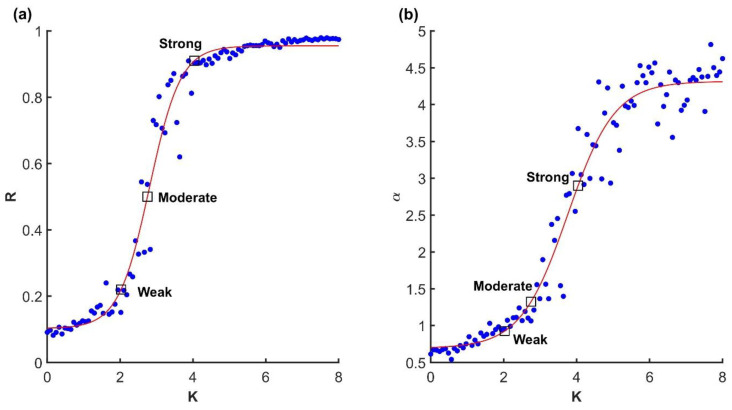
(**a**) Relationship between coupling strength *K* and synchronization (order parameter, Equation (4)) *R* in the finite size Kuramoto system with *N* = 100. (**b**) Relationship between coupling strength and variance scaling. The squares correspond to the three representative scenarios illustrated in Figure 1. Dots are trials and red lines are fitted sigmoid functions.

To keep the system commensurate in size with the empirical gait dataset presented below, we simulated the system specified by Equation (6) with N=100, σ=2 over 100 trials and varied K∈[0,8] uniformly across trials (see Figure 2a). In each trial, the distribution of intrinsic frequencies $\omega_{i}$ was drawn from a Gaussian distribution $N\left(0,\gamma \right)$ with γ=0.25. Each trial had a simulated duration of 100 s at a sampling rate of 300 Hz. We used a third-order Runge–Kutta solver. Note that the theoretical critical K cannot be determined exactly in a finite size model.

#### Analysis

PCA was performed on the real component of the phase oscillators. We used singular value decomposition after zero-centering each variable. The eigenvalues of each principal component were converted to variances in the range from 0 to 100 percent. We fitted a power law of the form $s_{n}\propto n^{-\alpha }$ (Equation (1)) to the rank-ordered variances (see Figure 1d–f). To remove the drop off region, we discarded variances below a fixed threshold of 0.1%. We also fitted a Zipf–Mandelbrot power law, which has two more parameters, but it appeared not to fit the curves as well.

### 2.2. Human Gait Recorded with High-Density Motion Tracking

For the human movement data, we used a publicly shared dataset [39] with full-body motion capture of participants performing short walking trials (six meters in a straight line).

#### 2.2.1. Study Population

The participants included healthy adults and patients with unilateral hip osteoarthritis (OA). Patients were recorded twice: once before hip-replacement surgery and again six months after successful surgery. The asymptomatic group included 80 healthy participants between the ages of 25 and 82. There were 106 patients with hip OA without other diseases between the ages of 45 and 85.

#### 2.2.2. Protocol and Equipment

Participants walked back and forth for six meters in a straight line at a self-paced speed. Each condition was recorded for a minimum of ten trails. Eight optoelectronic cameras sampled at 100 Hz (Vicon MXT40, Vicon, UK) and thirty-five reflective cutaneous markers placed on anatomical landmark locations were used to record 3D kinematics, resulting in a total of 105 position time-series variables.

#### 2.2.3. Analysis

For pre-processing, data quality was controlled by removing bad trials with markers missing for more than 50% of the duration of the trial. The positional drift due to forward translation while walking was removed by regressing out the translation. To equalize data length across trials, participants, and groups, each trial was re-sampled to 500 time points using linear interpolation, so that there were roughly 100 samples per second.

The variance spectrum scaling analysis followed the procedure described in the Analysis in Section 2.1. The only difference was that the cutoff threshold was set to a lower value, 0.01%. Finally, the inherent dimension was estimated using two methods suitable for nonlinear manifolds [24]. The fractal dimension was estimated with the correlation dimension method [40]. The intrinsic dimensionality was estimated with a maximum likelihood method [41]. These were computed with the Matlab toolbox for dimensionality reduction (https://lvdmaaten.github.io/drtoolbox/, version 0.8.1b, accessed on 19 March 2025).

## 3. Results

### 3.1. Variance Scaling in the Kuramoto System

To confirm that the Kuramoto model was valid, and that the critical transition region of parameter space was located correctly, we first focused on synchronization as a function of coupling strength, as shown in Figure 2a. Indeed, the relationship between the order parameter *R* and coupling strength *K* exhibited the familiar sigmoid shape, although not with a sharp transition from 0 to 1 as expected from the ideal model.

Next, we consider variance scaling at selected points in parameter space. As expected, we found that when the model was in the higher end of the critical region, variance scaling crossed α=3, as shown in Figure 2a,b, which is the threshold of smoothness for d=1, Equation (2). We assumed that manifold was d=1 because, by definition, the Kuramoto model in its coherent state attracts all units to one synchronized oscillator. In short, when coupling is set just above the critical value, the Kuramoto model exhibits critical balance between a globally synchronized and smooth manifold and the distribution of variability among its embedding dimensions.

### 3.2. Variance Scaling in Human Gait

**Figure 3:**
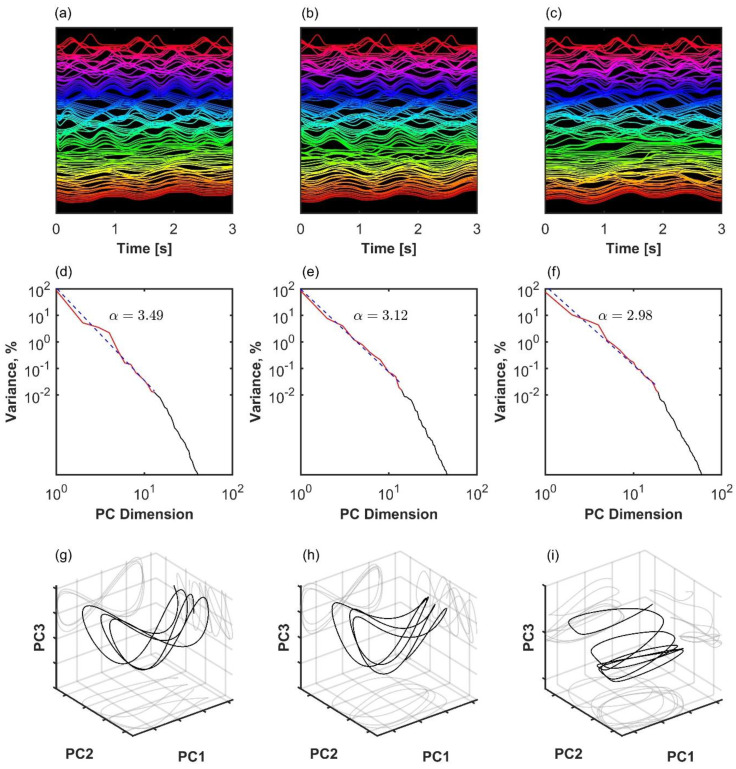
Three examples of coordination analysis in the human kinematic gait data with *N* = 105 markers. (**a**–**c**): Time series of kinematic markers. For better visibility, a short time window is shown, and time series are normalized with the z-score and shifted on the *y*-axis. (**d**–**f**): Eigenspectrum scaling computed by fitting an inverse power law $s_{n}\propto n^{-\alpha }$ to the variance in principal components. (**g**–**i**): The first three principal component projections. Left column (**a**,**d**,**g**): healthy participant with a high scaling exponent. Middle column (**b**,**e**,**h**): patient after successful arthroplasty exhibits slower decay in the variance spectrum, meaning that there is more variance distributed among the less important dimensions of the manifold. Right column (**c**,**f**,**i**): patient before arthroplasty exhibits even slower decay in the variance spectrum. In the latter case, scaling approaches the theoretical lower bound for smooth manifold with *d* = 1, Equation (2).

**Figure 4:**
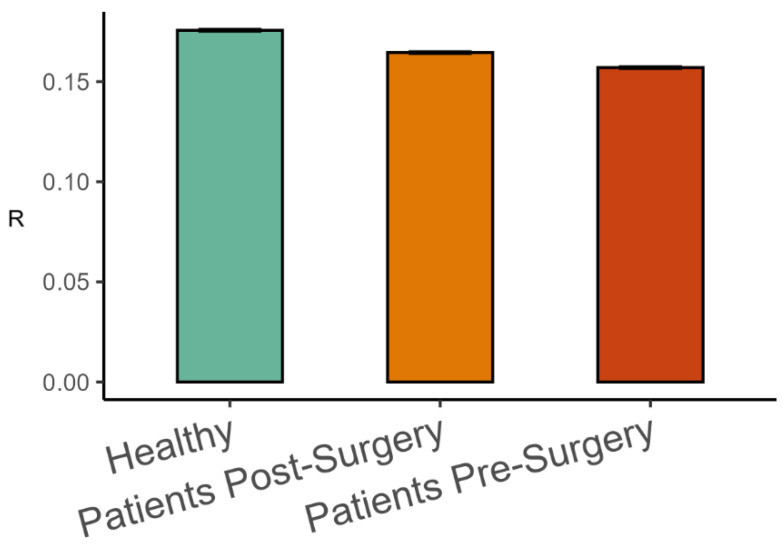
Mean phase synchronization among body-worn markers (Mean ± 95%CI) in the three groups of participants.

**Figure 5:**
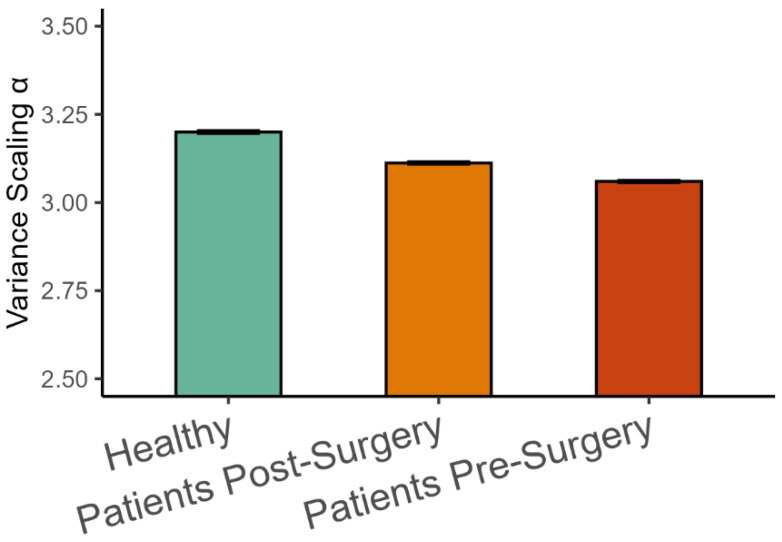
Variance scaling exponents (Mean ± 95%CI) in the three groups of participants.

The analysis of representative trials from each group is illustrated in Figure 3. The statistical analysis with a linear mixed-effects model showed that phase synchronization among kinematic degrees of freedom, Equation (4), was higher in healthy participants than in patients after surgery (β = 0.011, *SE* = 0.001, *p* < 0.001, Cohen’s *d* = 0.649), and it was higher after surgery than before surgery (β = 0.008, *SE* = 0.001, *p* < 0.001, Cohen’s *d* = 0.496), as shown in Figure 4. Importantly, the highest scaling in the variance spectrum was observed in the healthy participants, followed by patients after successful surgery (β = −0.318, *SE* = 0.047, *p* < 0.001, Cohen’s *d* = 0.680). Scaling in patients before arthroplasty surgery was lower than after surgery (β = −0.108, *SE* = 0.013, *p* < 0.001, Cohen’s *d* = 0.230). As expected, all groups were close to but above the critical value of α=3 on average (see Figure 5). Interestingly, the pre-surgery group was closest to the critical value.

**Figure 6:**
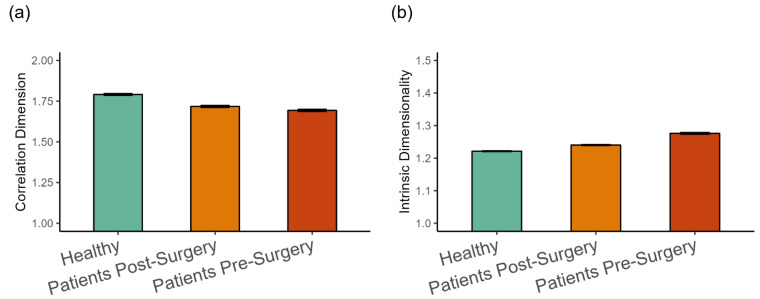
(**a**) Fractal dimension estimated with the correlation dimension method, and (**b**) intrinsic dimensionality (Mean ± 95%CI) in the three groups of participants. Intrinsic dimensionality was estimated with an MLE method.

The dimensionality analysis revealed that, on average, the kinematics of walking was in the range between one and two dimensions (see Figure 6). The fractal dimension was highest in the healthy group, followed by patients after surgery (β = −0.077, *SE* = 0.021, *p* < 0.001, Cohen’s *d* = 0.312), and then by patients before surgery (β = −0.021, *SE* = 0.08, *p* < 0.001, Cohen’s *d* = 0.084), as shown in Figure 6a. This indicates a tendency for more complex patterns of movement in the healthy group. The trend was reversed for the intrinsic dimension (see Figure 6b). It was lowest in the healthy group, higher for patients after surgery (β = 0.021, *SE* = 0.009, *p* < 0.05, Cohen’s *d* = 0.265), and patients before surgery were higher than after surgery (β = 0.039, *SE* = 0.002, *p* < 0.001, Cohen’s *d* = 0.499).

**Figure 7:**
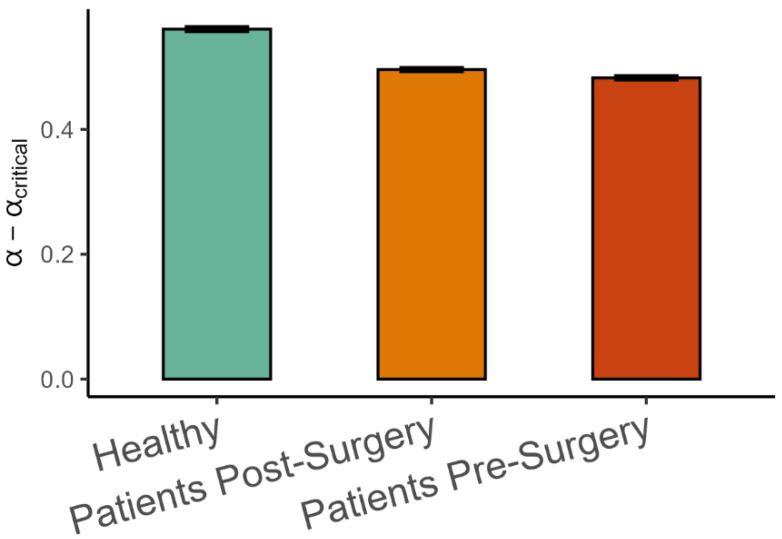
Mean (±95%CI) differences between observed variance spectrum scaling and the critical variance scaling in the three groups of participants. α_critical_ was calculated by using Equation (2) and substituting intrinsic dimensionality for *d*.

Finally, the difference between the observed variance scaling α and the critical α_critical_ estimated from the intrinsic dimension using Equation (2) is shown in Figure 7. As hypothesized, α was on the smooth side of the critical value, meaning that α > α_critical_, but the difference was lower in patients after surgery than in healthy patients (β = −0.066, *SE* = 0.014, *p* < 0.001, Cohen’s *d* = 0.45), and it was lower in patients before surgery than after surgery (β = −0.013, *SE* = 0.004, *p* < 0.001, Cohen’s *d* = 0.09).

## 4. Discussion

We introduced an approach to investigating properties of full-body movement manifolds, as they are embedded in much higher dimensional observation spaces comprising potentially hundreds of kinematic markers. The rate of decay in the eigenspectrum (variance spectrum) after dimensionality reduction is related to the smoothness of the manifold and the distribution of coordination variability among different degrees of freedom. We defined a reference value, Equation (2), that enables the principled interpretation of the observed results in terms of boundary and optimality conditions.

The ideas were confirmed using the Kuramoto model, a system of phase oscillators with various amounts of phase-locking between them and a critical region of coupling strength. We observed that the super-critical regime was associated with a balance between the smoothness of the manifold of the globally synchronized system and distribution of variance among the dynamic units.

We then studied whether full-body movement organization during walking was characterized by a similar regime. Healthy walking was characterized by a low intrinsic dimension in the range between one and two and variance spectrum scaling corresponding to a smooth manifold. With increasing gait impairment, namely in patients after surgery and patients before surgery, the intrinsic dimension increased and variance spectrum scaling decreased. This can be interpreted as an overall reduction in the coordination among degrees of freedom in this full-body task. With impairment, the scaling came closer to the critical threshold for non-differentiability, but it did not cross it. It should be noted that the patients in this study, while in pain, were capable of independent ambulation. It is possible that a more severe gait disorder will manifest as a pathological manifold with non-differential singularities. Interestingly, the correlation dimension decreased with increasing gait impairment, contrary to the intrinsic dimension, which increased. While overall dynamics became less synchronized, it also became less complex with gait impairment.

The empirical data departed in some ways from the way in which the theoretical model accounted for the balance between synchronization and variance scaling. The Kuramoto model exhibited super-critical scaling when it was approaching global synchronization. In the empirical data, super-critical scaling was observed too, but overall synchronization was much lower. It is possible that this is related to the limited range of phenomena accounted for by this model. In its classical form used here, the Kuramoto model is well suited to account for the onset of global synchronization in a collective of coupled oscillators but not as much for metastability and multi-stability. The latter phenomena are associated with biological movement systems and various motor tasks [42,43]. This means that human gait can enclose a rich repertoire of dynamic behaviors while maintaining a small and smooth manifold.

The present is a minimally presumptive approach to investigating high-density coordination and it has the potential to inform classic issues in movement science. Elsewhere, the question of task and neural space dimensionality has generated a lively debate concerning the structure of the neural substrates supporting different activities [44,45,46,47,48,49,50,51,52]. In the context of neural coding, an efficient approach that preserves resources is one where information is uniquely coded in separate subspaces. On the contrary, a stable approach is one in which all degrees of freedom are involved redundantly as a neural population.

A similar question concerns the structure of multidimensional biomechanical variability supporting different tasks [53]. Historically, progress has been limited by expensive methods for the comprehensive tracking and analysis of movement. In fact, the movement sciences often deal with the problem of up-dimensionalizing few observed variables to uncover inherent system dynamics. A popular method for this is phase space reconstruction [54]. In contrast, we are now facing the opposite problem: high-density and highly redundant datasets require techniques to determine properties such as smoothness, dimensionality, synchronization, and complexity.

To help interpret the geometry of full-body dynamics, we pointed out that the manifold distribution and smoothness serve to define a reference scaling value by combining two constraints with opposite directions. This is based on biomechanical considerations. On one hand, the smoothness condition considers the fact that sharp transitions in the manifold of positional coordinates would imply diverging velocities. It would be an inefficient control strategy to correct full-body movement by sharp sudden impacts, not to mention that infinite velocity is hard to make sense of in a mechanical system. Mathematically speaking, non-differentiable gait cycle dynamics imply instability, falls, and, generally, unpredictability. This is a tendency to increase the absolute slope of the singularity spectrum. On the other hand, distributing variability across a wider range of degrees of freedom is a strategy for flexibility and resilience to perturbations. This is a tendency to decrease the absolute slope of the singularity spectrum. The combination of both tendencies leads to optimal scaling in the vicinity of the smoothness threshold, Equation (2).

Here, we assumed that the task of walking is fundamentally low-dimensional but distributed among degrees of freedom. This can be motivated by the theoretical debate about whether bipedal gait can be reduced to a simple one-dimensional template model [55] such as an inverted pendulum [56,57], a swinging limb as a pendulum [58], etc. Plugging *d* = 1 in Equation (2) led to $\alpha_{critical}=3$, which was close to the empirical observation. It is possible, however, that this assumption oversimplifies gait dynamics. Empirically, we found that the threshold exponent was lower because the intrinsic dimension was higher than one.

Another theoretical advantage of addressing full-body coordination is that it avoids relying on weakly justified choices about which coordination pair is the relevant one. In some instances, relevance is implied by the task, as in swinging limbs to maintain a given rhythm for ambulation [59,60]. In free and full-body tasks, however, it is not always possible to instantiate the experimental conditions necessary to discover the relevant collective variables.

The present approach has further potential to inform classical ideas in movement science. The objective of exploring all dimensions of variability, whether they are obviously involved in the task or not, is mathematically related to the highly influential (un)controlled manifold framework [61,62]. Furthermore, we have yet to explore whether the optimal properties of the high-dimensional manifold relate to the ideas of the optimal variability and so-called biological variability of one-dimensional movement variables [63,64].

## Data Availability

No new data were created.
